# The Story of the Insane from Year to Year

**Published:** 1905-03-04

**Authors:** 


### THE STORY OF THE INSANE FROM YEAR TO YEAR.

SEVENTEENTH ANNUAL SERIES.

### SALOP AND MONTGOMERY ASYLUM.

ON the first of January this asylum contained 807 patients, and by December 31st the number had risen to 833. The average number resident was 790, and the total number under treatment was 1,006. Calculated on the admissions the recoveries stand at 29·56 per cent., and calculated on the average number resident the deaths were 13·61 per cent., the first of these being under the County Asylums average and the second above it. There were only 2 deaths from general paralysis of the insane, and the total from cerebral and spinal diseases was only 18, but we notice that 25 of the 111 deaths were caused by consumption and 12 by other lung diseases, pneumonia accounting for 9 of these. On the other hand the deaths table shows no case of colitis. Table 10 has the physical and moral causes of insanity mixed together, but as the insanity in 104 of the 199 admissions was of unknown origin the statistics are of little or no value. Neither the Committee of Visitors nor the medical superintendent comments on the high death-rate from phthisis. The asylum contains space for only 751 patients, so that it has been considerably over-crowded during the year, and to reduce this the Committee have decided to board out 20 patients at the Abergavenny Asylum, and have inquired as to the possibility of transferring patients to the workhouses. The first of these is an admirable temporary arrangement, but it ought never to have been necessary. The average annual increment of patients is a perfectly well-known quantity, and the asylum ought to have been enlarged in anticipation of it. In the meantime the 20 patients sent to Abergavenny Asylum will cost about £250 a year more than they would at Salop, and for this sum at least one bed-space could have been added to the asylum; and it is certain that the 20 boarded-out cases will soon have to be increased to 40 or more. As to sending chronic cases to workhouses it is an expedient unlikely to prove permanently successful to any great extent until the capitation grant has been adjusted, and there was no reference to this much-needed reform in the last Lunacy Acts Amendment Bill. Attendants and nurses are being trained for their duties by the assistant medical officers, Drs. Rigden and Downey, and several of the staff have passed the examination of the Medico-Psychological Association, a step which will, we trust, be merely a prelude to another examination subsequent to a full three years' course of instruction. The appliances for extinguishing fire have been over-hauled and outside staircases are being put up. Attendants Thomas Jackson and William Vaughan received pensions during the year. The rate for private patients is only 15s. a week and that for paupers is 9s.

